# One Health Integration: A Proposed Framework for a Study on Veterinarians and Zoonotic Disease Management in Ghana

**DOI:** 10.3389/fvets.2018.00085

**Published:** 2018-05-02

**Authors:** Sophie Françoise Valeix

**Affiliations:** Institute of Development Studies, University of Sussex, Brighton, United Kingdom

**Keywords:** veterinarians, ghana, one health, zoonoses, perspectives, practices, relationships, integration

## Abstract

In parallel with the recent world-wide promotion of One Health (OH) as a policy concept, a growing body of social science studies has raised questions about how successful OH policies and programs have been in managing some global health issues, such as zoonotic diseases. This paper briefly reviews this literature to clarify its critical perspective. Much of the literature on OH also is focused on health management at an international level and has paid less attention to implementation programs and policies for OH at the national and local levels, especially in low-and-middle-income countries (LMICs). Programs to implement OH often are linked to the concept of “integration”, a notion that lacks a universal definition, but is nonetheless a central tenet and goal in many OH programs. At the local and national levels, strong differences in perspectives about OH among different professions can be major barriers to integration of those professions into OH implementation. Policies based on integration among professions in sectors like animal, human and environmental health can threaten professions’ identities and thus may meet with resistance. Taking into account these criticisms of OH research and implementation, this paper proposes a research framework to probe the dominant social dimensions and power dynamics among professional participants that affect OH implementation programs at the local and national levels in a low-income country. The proposed research focus is the veterinary profession and one aspect of OH in which veterinarians are necessary actors: zoonotic disease management. Results from research framed in this way can have immediate application to the programs under study and can inform more expansive research on the social determinants of successful implementation of OH programs and policies.

## Some Critiques of One Health

Human health is a globalized societal concern subject to complex global governance. It is replete with “wicked problems” underpinned by complexity, uncertainty and competing goals that resist straightforward understanding and resolution (1). One such wicked problem is emerging and re-emerging zoonotic diseases or zoonoses^1^ which threaten the health of populations as well as economies, livelihoods, and even political regimes (2, 3).

The concept of One Health (OH) became popular as a new health policy framework in the first decade of the 21st Century, initially to manage emerging disease threats. OH can be defined as “*a generalised and flexible term that captures the will to address the complexities and interrelations that exist between human, animal and ecological health*” (4). The OH concept is not new. It developed from the term “one medicine”, which was coined by Calvin Schwabe in the 20th Century to signify the paradigmatic similarity between human and animal medicine and their mutual benefits (5). By 2004, the concept of OH was being promoted all over the world and significantly gained traction after the pandemics of SARS^2^ (2003) and Avian Influenza (2005–2007) when the need for more multifaceted approaches in zoonoses research, policy and management was widely recognized (6).

A growing body of social science studies has raised questions about how successful OH policies and programs have been or can be in managing some global health issues, such as zoonotic diseases. Some see OH as a “*fragmented intellectual project*” used by different actors for different outcomes (7). Indeed, while a powerful rhetoric of advocacy for OH was developing internationally, with OH being portrayed as a methodology, approach, movement, strategy, or paradigm shift, critical views of OH were also emerging (8, 9).

The concept of “integration” is embedded in many policies and implementation strategies associated with OH. Integration is a notion that lacks an agreed-to definition but is nonetheless a central tenet and goal in many OH program plans. From a social point of view, integration can be defined simply as “*a way of describing the established patterns of human relations in any given society*”, which does not imply that integration is either negative (implying conflict) or positive (implying order) (10). Integration can more usefully be defined as “*developments that determine connections of related diverse elements into the social whole, system, community, or other unit*” (11).

Many OH scholars treat integration as a positive goal, with more integration representing better organization of people in policies and actions around a particular OH goal (10). While there is no universal definition of “integration,”, papers on OH often use the word interchangeably with “collaboration”, and, to a lesser extent, with “cooperation” or “coordination,” generally with respect to actors belonging to the domains of animal, human and environmental health. Thus, collaboration, cooperation and coordination are all seen as elements of, or complementary to, integration (12, 13). For the professionals engaged in OH programs, integration may lead to financial savings through sharing costs or to “holistic” thinking, planning across organisational hierarchies and paradigms via transdisciplinary work and consensus (14). It may also result in inclusion of the OH concept in training programs in many disciplines (15) and in recognising and articulating the “*implications of uncertainty on, and potential conflicts between, human values and political processes*” involving human, animal and environmental health (16).

Implementation of OH can be challenging. To start with, achieving integration among program actors is not always straightforward:

*“…studies emphasize how the goals of collaboration and coordination are a good deal easier said than done. Professional competition, conflicting priorities, institutional inertia and myriad other factors in diverse contexts make the implementation of OH projects a major undertaking” (*7*).*

The majority of publications frame OH policies and programs as being international in scope, viewing zoonotic disease management, for example, as a problem best tackled through high-level international coordination (17, 18). Less attention has been paid to how the OH concept resonates in various national and local settings, beyond some broad recommendations on changing national health systems (19, 20, 21), and little has been published about how the global OH agenda fits into existing national or local structures and practices (22, 23). Yet, important components of OH, such as zoonosis management, are fundamentally determined by what happens at the national level (6, 17). As an example, the case of the 2014 epidemics of Ebola in West Africa emphasizes the critical importance of local health structures (24).

Hinchliffe (25) worries that emphasizing the global dimension of OH promotes narrow approaches to OH implementation that fail to account for the diversity within global health issues. Giles-Vernick et al. (26) and Coffin et al. (27) recommend that OH research consider a wide range of knowledges that people from different locations and lifestyles have developed and transmitted in local contexts, and that such OH research should focus on low-and-middle-income countries (LMICs). Health policy in LMICs, where resources are limited and the responsibilities for public health often are divided among various government sectors and non-governmental agencies, is poorly studied and requires special attention in relation to OH (6, 28).

Axelsson and Axelsson (12) propose that the biggest barriers to achieving integration in health programs are differences in values, cultures, interests and commitments among the principal actors, and these differences often are very specific to each particular profession. Significant differences of perspectives on OH issues and their solutions can be expected across the veterinary, medical and environment sectors (29).

Kingsley and Taylor (7) recommended studying integration through a systemic approach that takes complexity into account because policies and actions can be intertwined through non-linear complex systems and processes which are embedded in the dynamics of power and politics vis-a-vis policy making and implementation in local, national and global contexts (30). It has been recommended that these complex processes be studied in a development context with an ethnographic approach that captures the social dimensions underpinning interactions between multiple actors from various institutions (31, 32).

## A research framework to explore the social dimensions of One Health implementation

To address some of the criticisms of OH research and implementation, and in recognition of the potential utility of a better understanding of integration in the OH context, this paper offers a research framework to probe the dominant social dimensions and power dynamics among professional participants that affect OH implementation programs at the local and national levels in a low-income country. This framework is essentially the research plan for an on-going study of aspects of OH implementation in Ghana for which the results and analysis are not yet complete. The research focus is the Ghanaian veterinary profession and one aspect of OH policy and programs in which the veterinary profession is a necessary actor: zoonotic disease management. The overall objective of this research framework is to document how one professional group perceives and participates in management of zoonoses and the nature and dimensions of the relations veterinarians^3^ have with other professions and with each other.

Portrayed as one of the “historical One Health actors” and “super One Health professionals,” veterinarians already are dominant players in OH research internationally (13, 33–37). However, and particularly in LMICs, veterinarians generally are in short supply, are under-resourced, and their contributions to public health are underestimated (35, 38). There is little in the literature on the complexities of the context in which the veterinary profession operates and strives to achieve OH outcomes in policy and practice. An understanding of the roles veterinarians play among other stakeholders in managing zoonotic diseases and how their roles might be enhanced at a national and local scale in LMICs is a missing piece of the information with respect to integration in OH.

Situated on the Gulf of Guinea in West Africa, a “hotspot” for zoonotic disease emergence (39), Ghana is a low-income country in which zoonotic disease risk is of substantial concern (40, 41). Ghanaian veterinarians have been involved in research on emerging and endemic zoonoses and in programmes that emphasize the need for integration in zoonosis surveillance and control, such as the WHO/AFRO strategy for Integrated Disease Surveillance and Response (1998); the assessment of the Ghanaian veterinary services by the OIE-PVS^4^ (2011); and the creation of the Ghana Field Epidemiology and Laboratory Training Programme, FELTP (2007). Nevertheless, Ghana does not have an organization, government department, or official plan with a clear mandate to pursue OH.

To identify the social dimensions influencing integration of veterinarians and other relevant professionals within policies and programs for zoonotic disease management in Ghana, this research framework proposes data collection on three main topics. The first is differences in professional** perspectives** on zoonoses associated with the variability of how knowledge and expertise are shaped in different branches of the contemporary natural sciences (42). The second is the professional **practices** by which veterinarians engage with the OH concept, with each other and with other professionals, and through which the concept of OH is transformed into field activities (43). The third is the **relationships** between veterinarians and other relevant professionals such as physicians and environmental scientists. These relationships are critical factors in the dynamics of integration and are the medium through which veterinarians as well as other professionals must broker and translate their technical knowledge to other actors in networks (31).

Thus, the proposed research framework provides an approach to the study of OH integration in terms of how veterinarians, as a professional group, think about their role(s) (perspectives), practically manage (practices) and interact (relationships) in their day-to-day routines in relation to zoonotic disease management in a LMIC. This research framework thus seeks to determine how veterinary perspectives, practices and relationships concerning policy and action around zoonoses can influence the scope of One Health integration.

## Concept 1: Perspectives

**Background:** OH integration requires that actors from different professions work together towards common OH goals, but professions differ in their perspectives on why and how to apply the OH concept operationally.

Analyses of the political economies of epidemics have shown that zoonoses and the associated policy responses to them may be understood differently by people and institutions with different interests and priorities (44, 45). Thus, different, and potentially contradictory, framings and agendas can create tensions regarding the implementation of OH in specific contexts. Competing narratives which called for different sets of policy responses have been noted in disease outbreaks (45). Differences in perspectives about zoonoses also are manifest in differences between international and local discourses (46).

While veterinary perspectives on health and disease management are expected to differ from those of other professionals (29), very few studies have carefully examined how in-country scientists or other professionals embedded within a national context frame zoonoses and OH (47, 48). Professional perspectives also can interfere with OH integration policy processes through divergent framings among disciplines (30, 49). For example, new acute zoonotic diseases potentially leading to pandemics may compete as priorities with endemic zoonoses in LMICs where both are present (32). Furthermore, dealing with major outbreaks through emergency-oriented and short-term interventions can occur at the expense of long-term, crucially-needed, health measures in LMICs (50).

**Research framework:** The data that will be most informative regarding veterinary perspectives on OH will be those documenting (1) the values and interests of veterinarians as a profession, (2) competencies attributed to veterinarians, including technical skills and knowledge, (3) historical evolution of veterinary institutions and attitudes toward these institutions and (4) effects of these veterinary perspectives on veterinary integration in zoonotic disease management programs.

To a large extent, professional values and interests derive from a profession’s notion of professionalism. During acquisition of professionalism, people select particular sets of values, orientations and beliefs (51) which evolve along with changes in society (52). Professionalism also represents a form of social control, based on processes of inclusion and exclusion of individuals among categories within a bureaucratic structure according to recognized qualifications and standards (51, 53). For veterinarians, this professional control can be exerted, for example, via veterinary associations (51). In Ghana, animal health services can be delivered by practitioners with different qualifications: veterinary surgeons, technicians, and community animal health workers (54). Elsewhere, the roles of these different kinds of veterinary service providers have been shown to overlap and to create tensions (55, 56). Thus, inquiry into veterinarians’ views of their own professionalism offers an entry point for documenting veterinary perspectives.

Inquiry into veterinary competences is another point of entry for research (57). Such competences entail both systems and interdisciplinary thinking, and the development of highly technical skills for multi-species health and illness through veterinary training (58–60). Data on how veterinarians define their professional competences, whom they view as having them and whom not, and how this differs among different veterinary employment scenarios will be key to assessing the social dimensions of OH integration.

Galaz and colleagues have written that perspectives on integration in OH have been driven by “*the legacy of each profession’s embedded histories*” (45; 61). For example, when veterinary medicine was institutionalized in Ghana, government veterinarians were within the Ministry of Health. After Ghana’s independence in 1957, they were transferred to the Ministry of Food and Agriculture (MoFA) (62), a change that very likely influenced veterinary perspectives regarding OH. Elsewhere in Africa, the veterinary profession has been shaped by institutional reforms such as decentralisation and privatisation (63–65) which likely also contribute to current veterinary perspectives. In this research framework, information on the history of veterinary institutions in Ghana - governmental, educational and professional - is to be gathered to serve as essential background information for evaluating current veterinary perspectives on OH.

## Concept 2: Practices

**Background:** While some research has examined the influence of OH concepts on professional practices (66), the influence of routine professional practices on the implementation of OH programs at local and regional levels often has been overlooked in the literature (23, 67). Hamilton (68) argues that looking at the importance that “material things” play in practices helps explain veterinary attitudes on the ground; in an ethnographic study of British farm veterinarians, she showed that material items, like faecal samples, were linked to particular meanings and to prestige differences within veterinary teams in which people had different qualifications.

Veterinary practice often includes considerable discretion, practitioners acting in ways that do not strictly fit official procedures in order to privilege certain interests, be they their own personal gain, the interests of certain clients, or public health interests (69–71). Discretionary behaviours in veterinary practice could either favour or impede veterinary integration in zoonosis management by implementing national guidelines versus emphasising local needs which may contradict these guidelines. As street-level bureaucrats (SLB), veterinarians often must find intermediary positions between compassion and flexibility that comes with caring for the circumstances of their local context, and impartiality with its rigid application of orders coming from top managers (69; Hasenfeld, 1992 in 71).

The notion of street-level discretion is generally portrayed as a negative factor that undermines policy implementation. Some recent studies, for example, have revealed resistance by local stakeholders, for socio-economic reasons, to cooperating in surveillance operations for avian influenza, which thereby limited the detection of avian influenza cases (38, 72). However, other studies of local health practitioners as SLB suggest a more positive impact of discretion vis-à-vis policy implementation. For example, Axelsson and Axelsson (12) argue that SLB in public health are likely to “*identify more with their clients than with their parent organisation*” and that the clients thus empowered represent opportunities for bottom-up policy integration. (73 showed that medical doctors in rural South Africa used discretion to “*align their practices with policies*” and “*compensate for inefficiencies and failures … in how the system functioned*”.

Zoonotic disease management is based on animal disease surveillance programmes aimed at early detection of zoonotic pathogens in domestic and wild animal populations in order to prevent outbreaks in humans. Public veterinarians^5^ are key actors in this surveillance, which involves continuous monitoring of the health of human or animal populations and evaluation of associated disease risk factors (74). The success of surveillance operations at the local level depends on the active collaboration of multiple stakeholders on the ground, such as veterinarians, farmers, traders and abattoir workers.

**Research framework:** The key research approach proposed to study current veterinary practices in Ghana and their influences on veterinary integration in zoonotic disease management is an observation of the routine practices of a sample of veterinarians. Important areas of inquiry within this observation include (1) the veterinarians’ interactions and views of the material components of their practices, (2) practitioners’ identification of, and values attributed to, major veterinary competences and different levels of training of veterinary service providers, (3) characterisation of veterinarians’ discretionary judgements and actions and the effects these may have on practices and on practitioners, and (4) the alignment or otherwise of current practices with effective zoonotic disease surveillance and potentially other forms of disease management.

## Concept 3: Relationships

**Background:** Relationships between OH actors, such as policy-makers, practitioners and researchers, in professional networks are a key dimension of the power dynamics at play in policy processes (30). Studies of professional relationships and OH generally have targeted large international networks (75) or international research activities (13, 37). Very few papers on OH have examined how interdisciplinary, cross-sectoral and inter-professional relationships actually work to advance OH integration in OH programs, particularly at a local or national level.

Work relationships are a form of social capital, which can be defined as “*the sum of the actual and potential resources embedded within, available through, and derived from the network of relationships possessed by an individual or social unit*” (76). Social capital can lead to collective action (77) and to integration of knowledge across organizations (78). Therefore, social capital is a useful window through which to study inter-professional relationships in the context of OH.

While social capital has been categorized according to various typologies, such as structural, relational and cognitive (76), or opportunity, motivation and ability-related dimensions (79), two main aspects of social capital cut across these typologies and are particularly relevant to the analysis of relationships in regard to veterinarians: (1) the social network and its structure, and (2) the potential benefits and assets mobilized through that network (76).

Social capital may favour or impede integration of veterinarians into zoonotic disease management. On the one hand, research has shown that relationships across sectors can facilitate collaboration on common issues even in the absence of formal structures and platforms for such collaboration. Inter-sectoral policy integration requires relationships which involve rational dialogue and mutual agreement (80). This link between dialogue and inter-sectoral collaboration has been picked up in the literature on integration in healthcare practice and is imbedded in the notions of “mutual adjustment” (81) and “power-sharing” (82). Vandersmissen and Welburn (75) consider “soft governance,” which relies on self-organisation in networks independent of control through hierarchy or legislation, to underpin OH, and Glouberman and Mintzberg (81) write:

*“(The notion of networks) suggests the linking together of interdependent organizations in all kinds of ways; to foster better communication in order to solve mutual problems. In between the authority of the hierarchy and the competition of the market sits the network of mutual relationships.”*

All these positive views of social capital express the idea that, assuming a good level of communication and trust exists between different health professionals, networks will spontaneously organise and engage actors in collaborative practices.

On the other hand, relationships in OH may not be associated with positive outcomes for integration. Binot et al. (13) remarked that long-term and collaborative relationships between veterinarians and professionals in other sectors, like agriculture, rural development or the environment, were insufficient globally. In the literature on health systems, inter-professional relationships often are presented as tense negotiation processes among different sectors’ interests through sectoral advocacy (83), with relationships framed as competitions in which power dynamics do not facilitate collaboration. In such advocacy coalition framing, relationships are competing networks of alliances. In conflicts over policy issues, the most powerful coalition will decide rather than decisions being based on a consensus achieved through collaboration (84).

**Research framework**: To explore the social capital present in the relationships of veterinarians among themselves, with other professionals, and with any other people or groups, data are needed that qualify and quantify (1) the social networks of veterinary relationships that exist and (2) the positive and negative impacts of these relationships on the OH focus of this research framework: zoonotic disease management. The first aspect, the network and its social structure, can be approached by determining (a) the presence or absence of relationships and how actors are connected, and (b) which relationships function within veterinary social networks, why they function as they do, and at what frequency of interaction (85, 86). Relationships with frequent interactions between veterinarians and other professionals associated with zoonosis management are an important focus for study because these may offer particular insight into the scope for OH integration in Ghana (13, 48, 87, 88).

The second aspect requires assessment of the quality of relationships (hostile or positive) in terms of facilitating or impeding actions and achievements which depend on these relationships. What do veterinarians’ relationships with other professionals mean to the veterinarians themselves and what assets do they represent vis-à-vis potential collaboration in zoonosis management? Data are required on how veterinarians maintain relationships with other professionals and whether these relationships are based on trust and reciprocity (77).

## Conclusion

This paper explores recent criticisms of OH implementation and the theoretical foundations for research on the notion of integration in OH. To respond to concerns that much of the research on OH has targeted international programs aligned with the priorities of wealthy nations, a research framework is proposed that targets implementation of OH at local and national levels in a low-income country. The proposed research framework offers an approach to qualifying and quantifying the social dimensions of OH implementation by investigating the professional perspectives, practices and relationships of veterinarians associated with their roles in zoonotic disease management in Ghana.

## Author Contributions

SV designed and implementated the research as well as wrote the manuscript.

## Conflict of Interest

The author declares that the research was conducted in the absence of any commercial or financial relationships that could be construed as a potential conflict of interest.

## Footnotes

^1^Zoonotic diseases can be defined as infections naturally transmitted between vertebrate animals and humans. Zoonotic agents are bacteria, viruses, fungi or other communicable disease agents (WHO). In 2008, zoonoses were shown to represent at least 60% of infectious human diseases and over 75% of emergent infections (39).

^2^Severe acute respiratory syndrome.

^3^In this paper, “veterinarian” is defined broadly to include people who have graduated (or are about to graduate) with a degree in animal health and are practising animal medicine and/or the management of animal diseases in an official government position or in private practices. This broad definition includes para-professionals, also known as technicians.

^4^Tool for the Evaluation of Performance of Veterinary Services developed by the World Organization of Animal Health (OIE).

^5^In Ghana, like in many other LMICs, most veterinarians work for the government and thus they work at the interface between citizens and the veterinary service bureaucracy.
